# Speed of sound in hydrogen isotopes derived from the experimental pvt data and an improved quantum law of corresponding state

**DOI:** 10.1038/s41598-020-58011-9

**Published:** 2020-01-22

**Authors:** Xiaojun Ma, Xing Tang, Zongwei Wang, Qi Wang, Dangzhong Gao

**Affiliations:** Laser Fusion Research Center, China Academy of Engineering Physics, Mianyang, 621900 China

**Keywords:** Acoustics, Thermodynamics

## Abstract

The speed of sound in hydrogen isotopes can be applied to accurately determine the density, virial coefficient and equation of state. The functional relation between the speed of sound in a real gas and the experimental PVT data is derived from the virial equation of states. Utilizing the relation, the speed of sound in n-H_2_ is calculated from the experimental PVT data available. The calculated results illustrate that the presented method has an accuracy of better than 0.25% within the pressure range of below 1500 atm. However, there is little experimental PVT data available for n-T_2_, therefore, an improved quantum law of corresponding state (IQLCS) method, which is based on the physical nature that the different virial coefficients represent the interaction between the different number of molecules, is proposed for obtaining the speed of sound in n-T_2_. Utilizing the IQLCS method, the speed of sound in n-T_2_ can be obtained from the available speed of sound data in n-H_2_ or n-D_2_ via scaling the corresponding fitting coefficients at same temperature and pressure. The simulated results demonstrate that the IQLCS method is more accurate than the classical law of corresponding state(CLCS) and the maximum deviation is about 0.52% over the pressure range of below 1500 atm.

## Introduction

Hydrogen isotopes have been extensively applied in modern industries especially for the controlled nuclear fusion such as inertial confinement fusion (ICF) and magnetic confinement fusion (MCF)^1^. The speed of sound in hydrogen isotopes is one of the essential thermodynamic properties, which can be applied to determine the universal gas constant, Boltzmann constant, virial coefficient, density and equation of state^2–4^. Moreover, it can also be effective to measure the fuel content of deuterium(D), tritium(T) or their mixture DT in the thermonuclear fuel container called as capsules, which is an isotropic multi-layered hollow shell with a diameter from 1 mm to 2 mm^5^.

Speed of sound in a real gas is one of the most accurate and measurable physical quantities. As long as the speed of sound is determined, the density and pressure of hydrogen-isotopes-fuel in the ICF capsule can be calculated conveniently from the measured speed of sound data^6,7^. Consequently, it is critically important to construct the functional relation, also referred to as acoustic viral state of equation, between the speed of sound and the pressure of hydrogen isotopes.

Owing to the widely applications of hydrogen isotopes in modern industries, particular attentions have been paid to the thermodynamic properties of hydrogen isotopes so that a large number of the theoretical and experimental speed of sound data of H_2_ and D_2_ are available. Hodge measured the speed of sound in hydrogen under pressure ranging from about 0~10Mpa utilizing the acoustic interferometer of the resonator^8^. Michels determined the PVT data of hydrogen and deuterium at pressure up to about 100Mpa^9^ and the speed of sound in hydrogen and deuterium were computed at the pressure up to about 250 MPa^10^. Liebenberg developed an empirical equation of state(EOS) for n-H_2_ and n-D_2_ from their measurements of PVT data in the range of about 200~2000 MPa^11,12^. Leachman calculated the speed of sound in parahydrogen from a new fundamental equations of state, and the corresponding data are accurate to within 0.5% in the pressure range of less than 100 MPa^13^. Chen proposed a new equation of state for hydrogen in a simplified form, where only the second virial coefficient is considered. The results obtained from their equation have a maximum error of 3.8% within the temperature range 173 K~293 K^14^. Garberoglio computed the second virial coefficient for molecular hydrogen, deuterium, and tritium employing path-integral Monte Carlo techniques and the results for H_2_ are consistent with the experimental data available^15,16^. Liebenberg deduced the speed of sound in tritium from their measurements in hydrogen and deuterium according to kinetic theory where ${c}_{{T}_{2}}/{c}_{{D}_{2}}=\sqrt{{m}_{{D}_{2}}/{m}_{{T}_{2}}}$ in the pressure range of 2~20 kbar, and the derived speed of sound have a deviation of about 1.1%^17^.

Unfortunately, the experimental speed of sound in hydrogen isotopes are still limited. Moreover, little experimental speed of sound and equation of states for tritium are available in the literature. Therefore, how to obtain accurately the speed of sound in hydrogen isotopes especially for the n-T_2_ is still a vital issue.

In this paper, an expression between the speed of sound in real gases and the PVT data was derived firstly based on the virial equation of state. Then an improved method, which is based on the fact that the different virial coefficient should have a different correlation coefficient, was proposed for deriving the speed of sound in n-T_2_ from the corresponding data in n-H_2_ and n-D_2_. The calculated results illustrated that both the proposed expression and the improved quantum law of corresponding state(IQLCS) method are accurate to within 0.52% over the pressure range of 0~1500 atm.

## Theoretical Analysis

### The relation between speed of sound and PVT data

The speed of sound *c* in a real gas can be written as a function of the pressure *p* and density *ρ* in the constant entropy process

1 $${c}^{2}={(\partial P/\partial \rho )}_{S}=1/{\beta }_{S}\rho $$

where ${\beta }_{S}=-\,{(\partial V/\partial p)}_{S}/V$ is the adiabatic compressibility, *V* is the molar volume.

The specific heat ratio *γ* can be given by

2 $$\gamma ={C}_{P}/{C}_{V}={\beta }_{T}/{\beta }_{S}$$

where *C*_*p*_ and *C*_*v*_ are the specific heat capacities at constant pressure and constant volume, respectively. ${\beta }_{T}=-\,{(\partial V/\partial p)}_{T}/V$ is the isothermal compressibility.

Substituting Eq. (2) into Eq. (1), the speed of sound can be written as

3 $${c}^{2}=-\,\gamma {V}^{2}/M{(\partial V/\partial p)}_{T}$$

where *M* is the molar mass of gas.

The equation of state in real gases can be given by

4 $$PV=RT(1+Bp+C{p}^{2}+D{p}^{3}+\ldots \ldots )$$

where *R* is the universal gas constant. *B*, *C* and *D* are the second,third and fourth virial coefficients, respectively.

Substituting Eq. (4) into Eq. (3), the speed of sound can be written as

5 $${c}^{2}=\gamma RT{(1/P+B+CP+D{p}^{2}+\ldots \ldots )}^{2}/M(1/{p}^{2}-C-2Dp-\ldots \ldots )$$

For the low-pressure gases, an equation truncated to three terms virial coefficients has an adequate accuracy for measuring the fuel content in the ICF capsule, where the pressure is about 0.5~10 MPa. The speed of sound can be reduced to

6 $${c}^{2}=\gamma RT{(1/p+B+Cp)}^{2}/M(1/{p}^{2}-C)$$

For an ideal gas, the virial coefficients can be considered as constants equal to zero, therefore, the speed of sound can be expressed as

7 $${c}^{2}={\gamma }^{0}RT/M$$

where the *γ*^0^ is the specific heat ratio of ideal gas.

Utilizing the Eq. (5), the speed of sound in n-H_2_ and n-D_2_ can be determined from the experimental PVT data.

The speed of sound in a real gas can be written as a function of the pressure *p* and temperature *T* in terms of the virial equation of state:

8 $${c}^{2}={\gamma }^{0}RT/M\cdot (1+B^{\prime} p+C^{\prime} {p}^{2}+D^{\prime} {p}^{3}\ldots )$$

where B′, C′ and D′ are the second, third and fourth acoustic virial coefficients of gas, respectively.

According to the theoretical and experimental speed of sound data in the literature, the speed of sound can be fitted as a function of pressure

9 $${c}^{2}={A}_{0}+{B}_{0}p+{C}_{0}{p}^{2}+\ldots $$

where A_0_, B_0_ and C_0_ are the fitted constants.

When only the second and third virial coefficient are considered, the acoustic virial coefficients can be obtained from the fitting coefficients by the following expressions

10 $${\gamma }^{0}RT/M={A}_{0}\,B={B}_{0}/{A}_{0}\,C={C}_{0}/{A}_{0}$$

Utilizing the Eqs. (5) and (9), not only can the speed of sound of real gases be determined from the experimental PVT data, the specific heat capacity, virial coefficients and equation of state can also be derived from the speed of sound.

### Speed of sound in n-T_2_

Utilizing the classical law of corresponding state(CLCS) method, the speed of sound in n-T2 can be obtained from the corresponding data of n-H_2_ and n-D_2,_ and the scaling relation can be given by^17^:

11 $${c}_{T}^{2}={c}_{i}^{2}{M}_{i}/{M}_{T}$$

where *c*_i_ is the speed of sound in n-H_2_ or n-D_2_, *M*_i_ is the molar mass of n-H_2_ or n-D_2_, *c*_*T*_ and *M*_*T*_ are the speed of sound and molar mass of n-T_2_, respectively.

Based on the Eqs. (8) and (9), the Eq. (11) can be rewritten as:

12 $$\begin{array}{rcl}{c}_{T}{(T,p)}^{2} & = & {M}_{i}/{M}_{T}[A(T)+B(T)p+C(T){p}^{2}+\ldots ]\\  & = & {\gamma }^{0}RT/{M}_{T}+{M}_{i}/{M}_{T}B(T)p+{M}_{i}/{M}_{T}C(T){p}^{2}+\ldots \end{array}$$

where A(*T*), B(*T*) and C(*T*) are the fitted constants at the temperature *T*.

Using the quantum law of corresponding state (QLCS) method, the reduced speed of sound in gases can be given by the following relation^18^

13 $${c^{\prime} }^{2}=M{c}^{2}/{N}_{A}\varepsilon $$

where *ε* is the well depth of Lennard-Jones(L-J) potential function, *N*_*A*_ is Avogdro constant.

It is noted that the reduced speed of sound in n-T_2_ is derived at the same reduced temperature and reduced pressure. The reduced density, reduced pressure and reduced temperature can be given by^13^

14 $$\begin{array}{ccc}\rho ^{\prime} ={N}_{A}{\sigma }^{3}\rho  & p^{\prime} ={N}_{A}{\sigma }^{3}p/\varepsilon  & T^{\prime} =RT/\varepsilon \end{array}$$

where *σ* is the inter-molecular distance with the zero potential energy.

From the Eqs. (13) and (14), the speed of sound in n-T_2_ can be predicted from the following relation

15 $${c}_{T}{(T,p)}^{2}=\frac{{M}_{i}{\varepsilon }_{T}}{{M}_{T}{\varepsilon }_{i}}{c}_{i}^{2}({\varepsilon }_{i}T/{\varepsilon }_{T},{\varepsilon }_{i}{\sigma }_{T}^{3}p/{\sigma }_{i}^{3}{\varepsilon }_{T})$$

According to the Eqs. (8), (9) and (15), the speed of sound in n-T_2_ can be expressed as

16 $$\begin{array}{rcl}{c}_{T}{(T,p)}^{2} & = & \frac{{M}_{i}{\varepsilon }_{T}}{{M}_{T}{\varepsilon }_{i}}[A({\varepsilon }_{i}T/{\varepsilon }_{T})+{\rm{B}}({\varepsilon }_{i}T/{\varepsilon }_{T})p+C({\varepsilon }_{i}T/{\varepsilon }_{T}){p}^{2}+\ldots ]\\  & = & \frac{{\gamma }^{0}RT}{{M}_{T}}+\frac{{M}_{i}{\varepsilon }_{T}}{{M}_{T}{\varepsilon }_{i}}B({\varepsilon }_{i}T/{\varepsilon }_{T})p+\frac{{M}_{i}{\varepsilon }_{T}}{{M}_{T}{\varepsilon }_{i}}C({\varepsilon }_{i}T/{\varepsilon }_{T}){p}^{2}+\ldots \end{array}$$

where $A({\varepsilon }_{i}T/{\varepsilon }_{T})$, ${\rm{B}}({\varepsilon }_{i}T/{\varepsilon }_{T})$ and $C({\varepsilon }_{i}T/{\varepsilon }_{T})$ are the fitted constants.

As we can seen from Eqs. (12) and (16), the speed of sound in n-T_2_ can be obtained from the available speed of sound data in n-H_2_ or n-D_2_ via scaling the corresponding fitting coefficients, where the second-order and high-order fitting coefficients are multiplied by a factor ${M}_{i}/{M}_{T}$ for the CLCS method or a factor ${M}_{i}{\varepsilon }_{T}/{M}_{T}{\varepsilon }_{i}$ for the QLCS method, respectively. Moreover, the first terms in the Eqs. (12) and (16) have the same form, which represent the speed of sound in the ideal gas. Noted that the speed of sound in n-T_2_ at the temperature *T* and pressure *p* need to be determined from the corresponding data in other hydrogen isotopes gases at the temperature ${\varepsilon }_{i}T/{\varepsilon }_{T}$ and pressure ${\varepsilon }_{i}{\sigma }_{T}^{3}p/{\sigma }_{i}^{3}{\varepsilon }_{T}$.

In fact, the speed of sound in a real gas can also be regarded as a function with the form of the virial equation, the first terms in both Eqs. (12) and (16) represent the speed of sound in ideal gas, which can be reduced to same form of ${\gamma }^{0}RT/{M}_{T}$_,_and the nature of other terms imply the intermolecular interaction. For instance, the second virial coefficient, which is the lowest order deviation from ideal-gas behavior, representing the interaction between pairs of molecules and the third virial coefficient meaning the interactions among three molecules. Due to the different physical nature of the virial coefficients with different orders, it may be a more reasonable that the different virial coefficient has a different correlation coefficient. For a pairs of molecules formed by two molecules, the correlation coefficient is ${\varepsilon }_{T}/{\varepsilon }_{i}$. Three molecules form three pairs of molecules, therefore, the correlation coefficient is ${({\varepsilon }_{T}/{\varepsilon }_{i})}^{3}$. The correlation coefficients of high order terms can be given by the expression ${({\varepsilon }_{T}/{\varepsilon }_{i})}^{n(n-1)/2}$ where *n* is the number of molecules.

Based on the consideration mentioned above, an semi-empirical relation of the speed of sound in n-T_2_ can be written as

17 $${c}_{T}^{2}=\frac{{M}_{i}}{{M}_{T}}({{\rm{A}}}_{{\rm{i}}}+{\varepsilon }_{T}/{\varepsilon }_{i}\cdot {{\rm{B}}}_{{\rm{i}}}\cdot P+{({\varepsilon }_{T}/{\varepsilon }_{i})}^{3}\cdot {{\rm{C}}}_{{\rm{i}}}\cdot {P}^{2}+{({\varepsilon }_{T}/{\varepsilon }_{i})}^{6}\cdot {{\rm{D}}}_{{\rm{i}}}\cdot {P}^{3}\ldots )$$

where subscript “*i*” means n-H_2_ or n-D_2_. *A*_*i*_ *B*_*i*_, *C*_*i*_ and *D*_*i*_ are the fitting coefficients of n-H_2_ or n-D_2_, respectively.

It is noted that the normal hydrogen isotope is a mixture of para- and ortho-hydrogen isotope at room temperature. For the normal hydrogen isotopes, the potential constants can be determined by linear interpolation

18 $$\begin{array}{c}{\varepsilon }_{n}=x{\varepsilon }_{ortho}+(1-x){\varepsilon }_{para}\\ {\sigma }_{n}=x{\sigma }_{ortho}+(1-x){\sigma }_{para}\end{array}$$

where *x* is the percent of ortho-hydrogen isotopes, ${\varepsilon }_{ortho}$, ${\varepsilon }_{para}$, ${\sigma }_{ortho}$ and ${\sigma }_{ortho}$ are the potential constants for para- and ortho-hydrogen isotopes, respectively.

For the normal hydrogen isotopes, the mixture concentration can be considered nearly as constant at the room temperature, for instance, the n-H_2_ or n-T_2_ has a mixture concentration of 75% ortho- and 25% para-H_2_ or -T_2_, and the n-D_2_ is a 2:1 mixture of ortho- and para-D_2._ Consequently, substituting the Eq. (17) into Eq. (18), the speed of sound in n-T_2_ can be calculated from the corresponding knowledge of n-H_2_ or n-D_2._

For the potential constants, Knaap and Beenakker found the following relation^13^

19 $$\begin{array}{c}\frac{{\varepsilon }_{ortho}}{{\varepsilon }_{para}}=1.006\\ \frac{{\sigma }_{ortho}}{{\sigma }_{para}}=1.0003\end{array}$$

Based on the Eqs. (18) and (19), the ratios of potential constants can be determined at different mixture concentration

20 $$\begin{array}{c}\frac{{\varepsilon }_{norm}}{{\varepsilon }_{para}}=1.004\,\frac{{\sigma }_{norm}}{{\sigma }_{para}}=1.0003\,(ortho:para=2:1)\\ \frac{{\varepsilon }_{norm}}{{\varepsilon }_{para}}=1.0045\,\frac{{\sigma }_{norm}}{{\sigma }_{para}}=1.00023\,(ortho:para=3:1)\end{array}$$

Because the differences of potential constants for the normal hydrogen isotopes with different mixture concentration are so small, the speed of sound in normal hydrogen isotopes can be determined directly from the corresponding data in other normal hydrogen isotopes.

## Results and Discussions

### Speed of sound derived from the experimental PVT data

**Figure 1:**
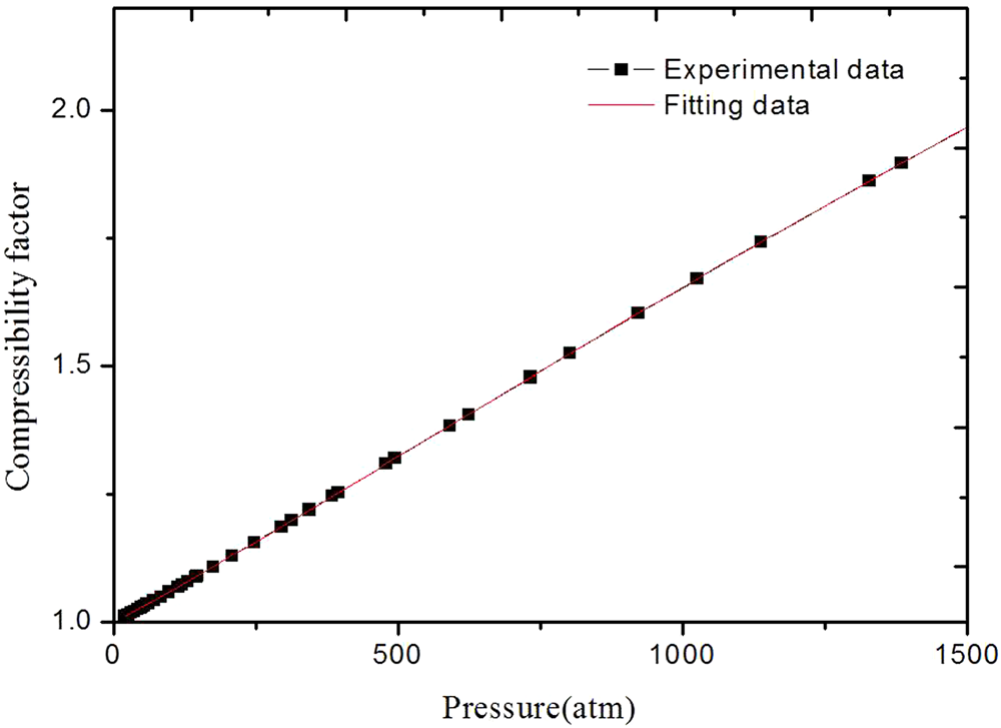
The experimental and fitting compressibility factor in n-H_2_.

In order to verify the accuracy of Eq. (5), the speed of sound in n-H_2_ are calculated from the experimental PVT data available. In this calculation, Michels’s data^9,10^ and the virial equation truncated to five terms are employed. In additional, the temperature applied in all calculations is 298.15 K. Figure 1 illustrates the compressibility factor of n-H_2_, plotted versus the pressure, over a range of 0~1500 atm. As we can seen in Fig. 1, there is a good agreement between the published and the fitting data obtained by the fifth-order polynomial fitting method. The maximum relative deviation is about 0.03% and the fitting coefficients are 0.99953, 6.00014 × 10^−4^, 1.65299 × 10^−7^, −1.66728 × 10^−10^, 6.58257 × 10^−14^, −9.75413 × 10^−18^, respectively. These results demonstrate that the fitting coefficients of n-H_2_ are accurate enough to quantify the compressibility factor in the pressure range of 0~1500 atm when the fifth-order polynomial are taken into account.

**Figure 2:**
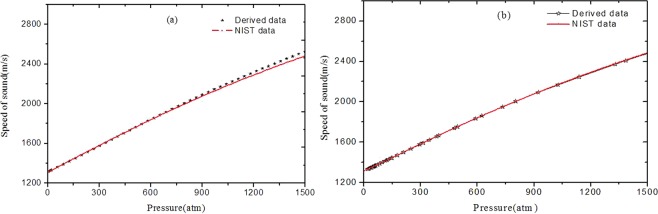
The speed of sound in n-H_2_ (**a**)without (**b**)with the specific heat ratio correction.

Utilizing Eq. (5) and the fitting coefficients, the speed of sound in n-H_2_ are obtained in the pressure range of 0~1500 atm. In this calculation, the specific heat ratio is considered as a constant of 1.41, the equation of state proposed by Leachman J. W., which is recommended by National Institute of Standards and Technology (NIST)^19^, is chosen to calculate the speed of sound in n-H_2_. The speed of sound calculated from Eq. (5) and the REFPROP software are shown in Fig. 2(a). It is to note that the calculated data from Eq. (5) are consistent with NIST data within the range of less than 600 atm, however, the deviations increase quickly with the pressure over the range of 600~1500 atm. It is well known that the specific heat ratio is dependent on the pressure and temperature. It is reasonable to consider the specific heat ratio as a constant in a limited pressure range. Nevertheless, the discrepancy from the approximation of specific heat ratio is not negligible in a large pressure range and this phenomenon, the deviations between the derived and NIST data increase quickly with the pressure in the high-pressure range, can be ascribed to the error of specific heat ratio at the different pressure.

In order to obtain the speed of sound with a higher accuracy in a large pressure range, the specific heat ratio with a variable of pressure is considered. The functional relation between the specific heat ratio and the pressure is obtained from Michels’s data by the polynomial fitting method. Figure 2(b) illustrates the speed of sound in n-H_2_ with the specific heat ratio correction. As can see in Fig. 2(b), there is an excellent consistency between the derived data and NIST data over the range of 0~1500 atm. The maximum relative deviation is less than 0.1% within the range of 0~900 atm and 0.2% within the range of 900~1500 atm.

### Speed of sound in n-T_2_ derived from the n-H_2_ and n-D_2_

In order to confirm the reliability of the CLCS, QLCS and IQLCS methods, the speed of sound in n-D_2_, which has adequate experimental and theoretical data available, are calculated firstly. In our calculation, the molecular constants are listed in Table 1.

**Table 1:** Molecular constants of hydrogen isotopes used in this paper^20^.

items	H_2_	D_2_	T_2_
M/(g/mol)	2.016	4.028	6.032
$(\varepsilon /k)/{\rm{K}}$	36.7	35.2	34.5
σ_/_(10^−10^m)	2.959	2.952	2.949

**Figure 3:**
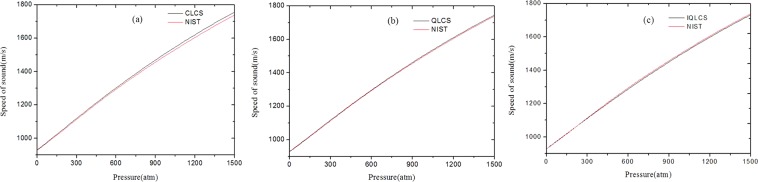
The speed of sound in n-D_2_ using (**a**) the classic law of corresponding states, (**b**) the quantum law of corresponding states and (**c**) the improved quantum law of corresponding states.

Figure 3(a) illustrates the speed of sound in n-D_2_, plotted versus the pressure. Compared with the NIST referenced data calculated from the equation of state developed by Richardson, I.A., an excellent agreement can be obtained in the pressure range of less than 300 atm. With the increase of pressure, the deviation between the derived data from the CLCS method and the NIST data is increasing. It is interesting that the speed of sound in n-D_2_ derived from the CLCS method are always lager than the NIST data with the increase of pressure. This phenomenon can be ascribed to the intermolecular interaction, which can be negligible only for the ideal gas. It can be seen clearly from Eqs. (8) and (12) that the CLCS method is based on the assumptions that the compressibility of n-H_2_ are equivalent to the corresponding values of n-D_2_ at the same pressure and temperature. Consequently, the CLCS method is applicable for the ideal or low-pressure gas where the intermolecular interaction is negligible and the compressibility can be regarded as constants. In fact, the compressibility strongly depend on the pressure, the higher pressure of gas means the more complicated intermolecular interaction. The approximation that n-H_2_ and n-D_2_ have equivalent compressibility at the same pressure and temperature may be inapplicable in the high- pressure gas. The calculated results show that the approximation is reasonable in the pressure range of less than 300 atm and the intermolecular interaction cannot be negligible in the high-pressure gas. Therefore, there is a rising deviation with the pressure increasing.

As shown in Fig. 3(b), the derived results from the QLCS method are similar to Fig. 3(a). Compared with the NIST data, there is an excellent consistency with a maximum relative deviation of 0.3% in the pressure range of less than 300 atm. The deviation in the high-pressure range of 300~1500 atm is slowly increasing and the maximum relative deviation is about 0.4%. It is noted that the speed of sound derived from the QLCS method is closer to the NIST data. One believes that the intermolecular interaction can also explain this phenomenon. Owing to the quantum characteristics of hydrogen isotopes, the effect of quantum mechanics on intermolecular interactions will lead to the deviation from classical molecular interactions. The QLCS method correct the intermolecular interaction using the well depth of L-J intermolecular potential function. The calculated results also demonstrate that the QLCS method improves the accuracy of speed-of-sound but there is still a small deviation especially in the high-pressure gas range.

Figure 3(c) illustrates the derived speed of sound in n-D_2_ from the IQLCS method, as we can seen in the figure, an excellent agreement with a maximum deviation of less than 0.2% can be obtained between the derived data and the NIST data within the pressure range of 0~300 atm. It is noted that the speed of sound in n-D_2_ are always smaller than the NIST data within the pressure range of 300~1500 atm and the maximum relative deviation is about 0.5%. The simulated results prove that the IQLCS method is more accurate than the CLCS and comparable with the QLCS methods.

**Figure 4:**
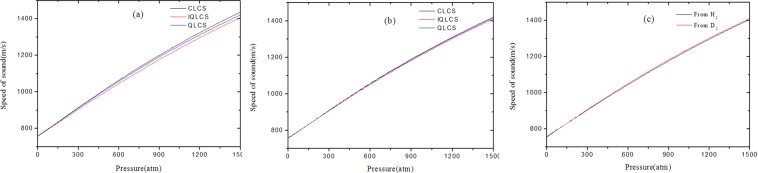
The speed of sound in n-T_2_ derived from (**a**) H_2_, (**b**) D_2_ and (**c**) comparison.

Figure 4(a) shows the speed of sound in n-T_2_ determined from n-H_2_ using the CLCS,QLCS and IQLCS methods. It is interesting that there are some similar characteristic as shown in Fig. 3(a,b). A good consistency can be obtained in the low-pressure range and the deviations increase slowly with the increasing of pressure in the high-pressure range. Moreover, the derived speed of sound from IQLCS is always less than the corresponding data calculated from CLCS method in the high-pressure range.

The speed of sound in n-T_2_ determined from n-D_2_ are shown in Fig. 4(b). Besides the similar properties as shown in Fig. 4(a), there are smaller deviations between the CLCS and IQLCS methods in the high-pressure range. One believe that the different intermolecular interaction between the hydrogen isotopes molecular will account for the phenomenon. As mentioned above, the CLCS method implies the approximation that the compressibility of hydrogen isotopes are regarded as constants at the same pressure and temperature. Generally, the difference of thermodynamic properties between the deuterium and tritium molecular are always smaller than the corresponding deviations between hydrogen and tritium molecular. Therefore, the derived speed of sound in n-T_2_ has a better consistency from n-D_2_ than from n-H_2_.

Figure 4(c) shows the speed of sound in n-T_2_ derived from n-H_2_ and n-D_2_ using the IQLCS method. The simulated results illustrate that the speed of sound in n-T_2_ derived from n-H_2_ and n-D_2_ are consistent and the maximum deviation is less than 0.2% over the pressure of 0~1500 atm. The good agreement between the speed of sound in n-T_2_ derived from n-H_2_ and n-D_2_ demonstrates that the IQLCS method is reliable.

**Figure 5:**
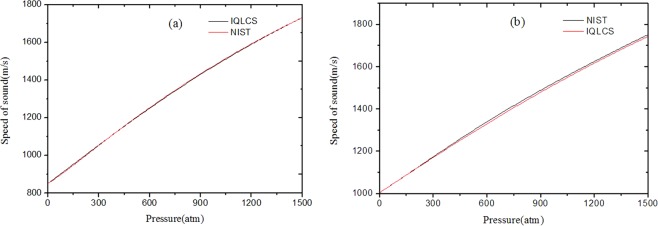
The speed of sound *c* in n-D_2_ using the improved quantum law of corresponding states at temperature 250 K (**a**) and 350 K (**b**).

Figure 5(a) shows the speed of sound in n-D_2_ determined from n-H_2_ at temperature 250 K using the IQLCS method. There is a good consistency between the predicted data and NIST data with a deviation of less than 0.3% over the 0~1500 atm range. The speed of sound in n-D_2_ determined from n-H_2_ at temperature 350 K are shown in Fig. 5(b), as we can see from this figure, the predicted data are consistent with the NIST data between the 0~300 atm range, and there are small deviations with a maximum deviation of about 0.5% between the 300~1500 atm range. The simulated results prove that the IQLCS method is reliable at different temperature.

### Comparison of calculated data to available data

**Figure 6:**
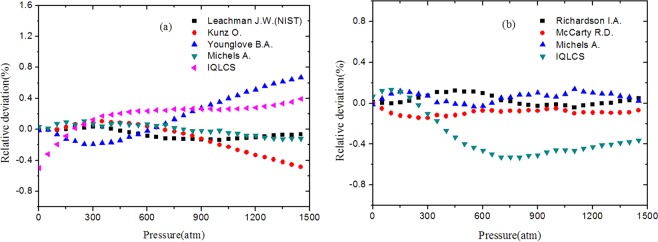
Comparison between the predicted speed of sound in n-H_2_ (**a**) and n-D_2_ (**b**) with IQLCS and others EOS.

To illustrate the accuracy of IQLCS, the speed of sound calculated from IQLCS are compared with the corresponding values predicted from the others EOS. In the comparison, the relative deviation is given by $({x}_{i}-\bar{x})/\bar{x}\cdot 100 \%$, where $\bar{x}$ is the average values of all calculated speed of sound from different EOS. The relative deviations of the speed of sound in n-H_2_ are illustrated in Fig. 6(a). As shown in this figure, the calculated data from IQLCS are consistent with the computed data from others EOS over the 0~1500 atm range, and the maximum deviation is about 0.49%. Figure 6(b) is the relative deviations of the speed of sound in n-D_2_. As we can seen from this figure, there is a good agreement between the values calculated with IQLCS and others EOS over the 0~1500 atm range, and the maximum deviation is about 0.52%.

In order to further confirm the reliability of IQLCS, the pressure of n-D_2_ are calculated from the speed of sound as an inverse problem of the Eq. (17). In this calculation, the absolute and relative deviations are applied to evaluate the discrepancy between the predicted pressure and NIST values over the range of 1~100 atm and 100~1500 atm, respectively. Table 2 list the pressure of n-D_2_ predicted from n-H_2_ at temperature 298.15 K. As shown in this table, there is a maximum deviation of about 2.1 atm between the predicted data and NIST data over the 0~100 atm range, and the maximum deviations is about 2.2% over 100~1500 atm range. The simulated results illustrates that the IQLCS method has an acceptable accuracy over 1~1500 atm. Compared with the predicted speed of sound, the calculated pressure have relative large deviations. This phenomenon demonstrates that the sensitivity coefficient of pressure, which can be considered as the ratio of the relative change in output quantity of pressure to the specific relative change in input quantity of sound velocity, is larger than the sensitivity coefficient of speed of sound.

**Table 2:** Comparison between the predicted pressure of n-D_2_ and NIST values.

Pressure		Deviation	
NIST/atm	Calculated/atm	Absolute/atm	Relative/%
1	0.3	−0.7	/
10	9.0	−1	/
30	28.6	−1.4	/
50	48.2	−1.8	/
100	97.9	−2.1	/
150	148.2	/	−1.2
200	199.3	/	−0.3
250	251.0	/	0.4
300	303.2	/	1.1
350	355.3	/	1.5
400	407.4	/	1.8
450	459.4	/	2.1
500	510.9	/	2.2
600	612.7	/	2.1
700	713.5	/	1.9
800	813.7	/	1.7
900	913.6	/	1.5
1000	1013.5	/	1.4
1100	1113.6	/	1.2
1200	1214.1	/	1.2
1300	1314.8	/	1.1
1400	1415.6	/	1.1
1500	1516.7	/	1.1

## Conclusions

In this paper, a functional relation between the speed of sound in a real gas and the experimental PVT data is derived theoretically based on the virial equation of state. A new method, which is applied to calculated the speed of sound in n-T_2_ from the available speed of sound data in n-H_2_ or n-D_2_ via scaling the corresponding fitting coefficients at same temperature and pressure, is proposed to improve the accuracy of the derived speed of sound. The speed of sound in n-H_2_ is calculated from the experimental PVT data available. The calculated results illustrate that an accuracy of less than 0.2% can be obtained within the range of 0~1500 atm.Using the CLCS, QLCS and IQLCS methods, the speed of sound in n-T_2_ are calculated from the speed of sound in n-H_2_ and n-D_2_. The calculated speed of sound data demonstrate that the IQLCS method can be applied to calculate accurately the speed of sound in n-T_2_ with a maximum deviation of about 0.52% over the range of 0~1500 atm.
